# Novel Treatments in Lupus

**DOI:** 10.3389/fimmu.2018.02658

**Published:** 2018-11-16

**Authors:** Milena Vukelic, Yi Li, Vasileios C. Kyttaris

**Affiliations:** Division of Rheumatology, Beth Israel Deaconess Medical Center, Harvard Medical School, Boston, MA, United States

**Keywords:** lupus, biologics, small molecules, treatment, clinical trails

## Abstract

Purpose of Review: The standard treatment options for systemic lupus erythematosus (SLE) are focused on non-specific immunosuppression. Over the past few years, scientific studies and ongoing clinical trials have shifted the paradigm with rapid advances in developing biologics and small molecules. A number of monoclonal antibodies and small molecule inhibitors have been developed to target specific pathways involved in SLE. Many of these novel therapeutic agents are already being tested in clinical trials and they may 1 day reshape the landscape of SLE treatment. Herein we review potential future therapeutic options for SLE.

## Introduction

In the past few years, greater understanding of the pathogenesis of SLE has translated into the development of more targeted therapeutic agents in various stages of clinical trials. Current treatment regiments for SLE typically comprise some combination of glucocorticoids, antimalarials, immune suppressive drugs, and cytotoxic agents in severe cases. The first biologic agent approved for SLE, Belimumab, has been in clinical practice for more than 5 years with overall positive albeit modest results (1). Therefore, developing more effective treatment for lupus remains a priority in the field.

**Figure 1:**
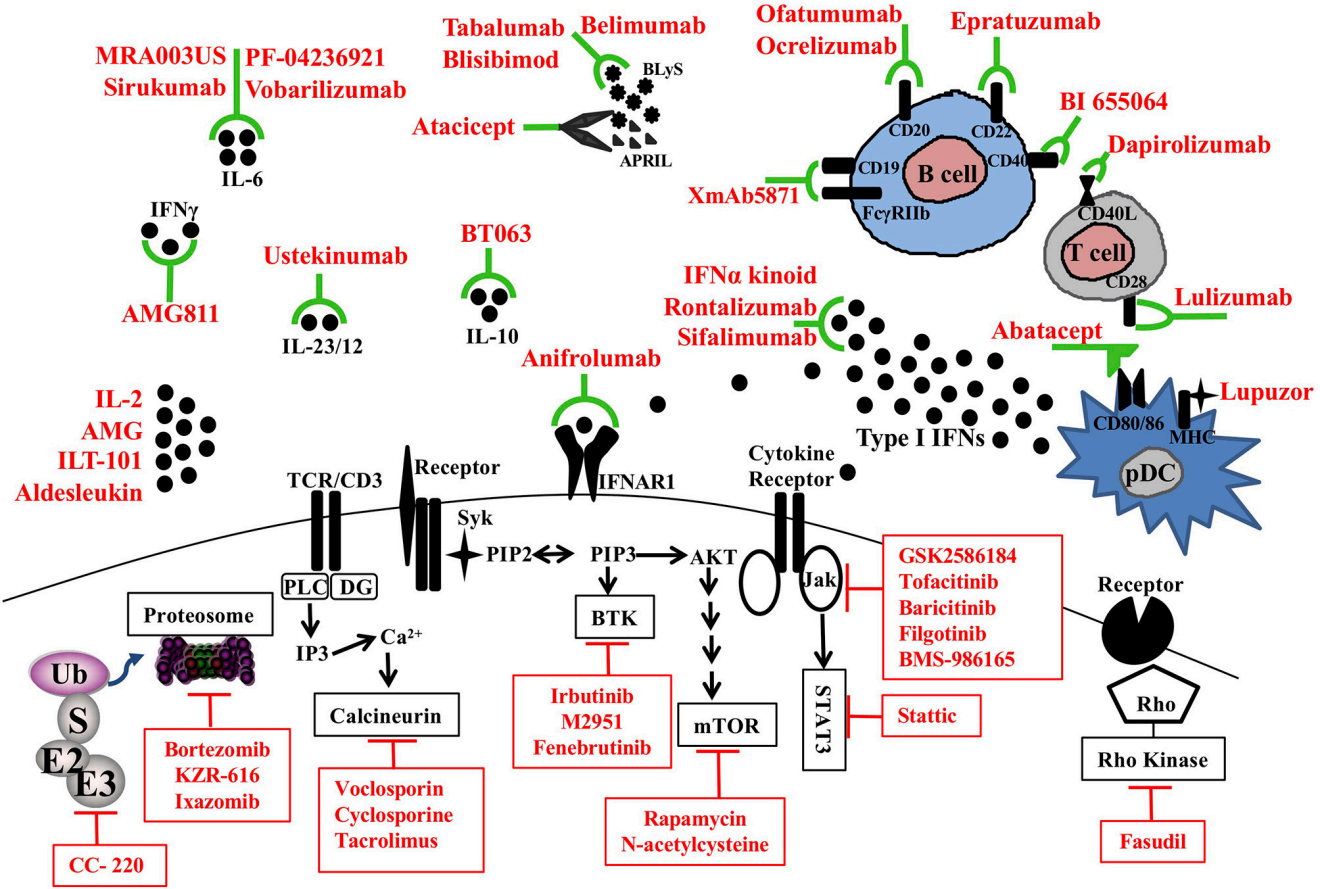
Therapeutic targets and novel treatments in SLE. Target molecules (black) and corresponding therapeutic agents (red) in clinical trials are illustrated. pDC, plasmacytoid dendritic cell; MHC, major histocompatibility complex; CD, cluster of differentiation; APRIL, a proliferating-induced ligand; BLys, B-lymphocyte stimulator; IL, interleukin; TCR, T cell receptor; IFNAR1, Interferon alpha receptor 1; BTK, Bruton's tyrosine kinase; mTOR, mammalian target of rapamycin; Jak, Janus kinases; STAT, signal transducer and activator of transcription proteins; Ub, ubiquitin; E3, Ubiquitin protein ligases.

Recent studies have identified numerous immunological checkpoints that are dysregulated in SLE and contribute to the loss of self-tolerance. A pipeline of novel agents are being developed to specifically target intracellular signaling pathways, inflammatory cytokines, chemokines, cell surface costimulation molecules, and the proteasome (Figure 1). Herein we will review the potential novel treatment options that are currently being tested in clinical trials for SLE.

### B cell inhibition

Systemic lupus erythematosus is a multisystem autoimmune disease characterized by the production of autoantibodies that primarily target a variety of nuclear antigens, deposit in tissues and activate complement. Plasma cells and their precursors, B cells, are fundamental to the development of these antibodies, and therefore are a prime therapeutic target for intervention in the disease.

B Lymphocyte Stimulator (BLyS)/A Proliferation-Inducing Ligand (APRIL) Belimumab was the first FDA approved fully humanized monoclonal anti-BLyS antibody for use in SLE more than 5 years ago. Administration of Belimumab was found to benefit SLE patients who had positive anti-double-stranded (ds) DNA and low complement (C3 or C4) levels. Moreover, the pivotal trials that lead to the approval of belimumab used a novel at the time composite outcome measure, the SLEDAI response index (SRI). Patients would be regarded SRI responders if they have (1) at least 4 points decrease in their SLEDAI scores over the period of the study, (2) No worsening of the physician global assessment (PGA), and (3). No new BILAG A or more than one BILAG B scores. The original SRI can be modified to require higher decrease in SLEDAI score e.g., by 5 or 6 points (SRI-5, SRI-6, respectively).

Compared to the placebo group's 44% SLEDAI response index (SRI)-4, the belimumab group had SRI-4 of 58%, indicating a statistically significant but yet modest effect. This modest effectiveness was later confirmed by an extensive post- marketing surveillance, as well as, its overall safety profile. Subcutaneously administered belimumab had similar efficacy at week 52 with SRI-4 response of 61.4% vs. placebo 48.4% (*p* = 0.0006) (2). It is worth mentioning that the effectiveness of belimumab remains unclear in severe renal and CNS disease as patients with these manifestations were excluded from the initial studies (1, 3). A study investigating the usefulness of belimumab in patients with lupus nephritis is currently ongoing (NCT01639339).

Tabalumab, a monoclonal antibody against BLyS that neutralizes membrane-bound and soluble BLyS, was assessed for its effectiveness in moderately active lupus in two large phase III clinical trials (ILLUMINATE-1 & 2). Although tabalumab treatment resulted in favorable changes in disease biomarkers (anti-dsDNA abs and complement levels), efficacy was marginal with SRI-5 of 38.4% in the tabalumab-treated vs. 27.7% in the placebo group (*p* = 0.002) in one trial. There was no statistical difference between the two groups in the second trial. Again, the treatment with tabalumab was found to be relatively safe as was the case with belimumab (4–6).

Blisibimod is a BLyS -neutralizing agent composed of a tetrameric BLyS binding domain fused to a human IgG1 Fc region. It binds both soluble and membrane-bound BLyS. In the recently completed phase III trial (CHABLISSC1) in patients with severe disease (SELENA-SLEDAI score ≥10), blisibimod showed a statistically significant steroid-sparing effect, reduction in SLE autoantibodies, B cell count, and proteinuria while increasing complement levels (7). SRI-6 as a primary end point was not met since response rate in the control subjects in this study was very high compared to prior SLE trials; 46.9% in the Blisibimod vs. 42.3% jn the control group. Higher steroid dosing in the placebo arm may have contributed to the relatively high response rates, confounding the primary efficacy outcome. Blisibimod was well-tolerated and the most common adverse events were upper respiratory or urinary tract infection and diarrhea (7).

Atacicept is a fully human recombinant fusion protein made of the extracellular portion of the TACI receptor and the Fc portion of human IgG. As atacicept blocks both BLyS and APRIL (8), it was predicted that atacicept may have a more potent effect on immunoglobulin production. Indeed, a significant high risk of severe infection and a decreased in immunoglobulin levels lead to a terminated phase II/III APRIL-SLE trial in nephritis (9). Similar effect on immunoglobulin levels was seen in the ADDRESS II trial (10) where effectiveness of atacicept to improve serologic markers and prevent lupus flares was superior to placebo only with 150 mg twice weekly dosing. The safety profile was acceptable with no reportedly increase in the overall frequency of serious adverse effects as compared to placebo. However, further assessment of the long-term safety of atacicept is warranted as this study only evaluated the safety and efficacy at 24-weeks (10). Given these results, the initial enthusiasm with this molecule has largely dissipated.

Overall, anti-BLyS but probably not anti-APRIL therapies, represent a moderately effective and safe approach in the management of patients with moderately active SLE with musculoskeletal and skin manifestations, especially if they remain corticosteroid dependent.

### Anti-CD 20

Unlike BLyS inhibition with the capacity of altering B cell maturation, CD20 targeting therapy depletes mature B cells without affecting plasma cells. Rituximab (RTX) is the most widely used anti-CD20 antibody; due to its chimeric nature, it was found to cause allergic reactions in approximately 10% of patients. Therefore, in the past few years several fully humanized anti-CD20 antibodies have been developed, such as ocrelizumab, ofatumumab, and obinutuzumab. Small uncontrolled trials showed that rituximab, already known to be effective in rheumatoid arthritis (11), can also ameliorate lupus (12). The non-randomized “Rituxilup” trial (*n* = 50) used rituximab and methyl prednisolone followed by mycophenolate mofetil in newly diagnosed lupus nephritis. Ninety percent of patients achieving a partial or complete remission by 37 weeks of treatement. A randomized multicenter clinical trial conducted by Rovin et al., was recently terminated prematurely due to slow recruitment (CTN84054592). But other pivotal trials in lupus nephritis (LUNAR) (13) and non-renal SLE (EXPLORER) were largely negative (14). Currently, the European League Against Rheumatism (EULAR) recommends rituximab as a treatment of last resort in severe lupus (15).

More recently, a few case reports (16, 17) and a phase 2A open-label proof-of-concept suggested that the combination of RTX and anti-BLyS (belimumab, BLM) could be effective. 11/16 patients with refractory SLE achieved renal responses (defined as proteinuria decreased to ≤0.7 g/24 h, normal serum albumin, stable creatinine and a normal urinary sediment). Importantly, RTX + BLM reduced nuclear autoantibody titers, and prevented the spike of circulating BLyS that is common after B-cell depletion. Similar results were observed in another small case series of patients with refractory SLE, who entered long term remission and discontinued corticosteroids (18, 19).

Similarly, the humanized anti-CD20 ocrelizumab failed to show significant efficacy in two early terminated phase III trials (BELONG and BEGIN). Patients with class III and IV nephritis were enrolled in BELONG (20) that compared ocrelizumab to placebo. Patients also received mycophenolate mofetil or cyclophosphamide (euro-lupus nephritis treatment protocol). Non-renal SLE patients were enrolled in the BEGIN trial. Both trials were terminated after significant increases of severe infections were noted in the ocrelizumab group. It has to be noted that efficacy analysis showed a trend favoring ocrelizumab over placebo.

Ofatumumab was administered to 16 SLE patients who could not tolerate rituximab; 87% (14/16) of these patients tolerated the infusion. About 85% patient achieved B cell-depletion with associated improvements in serological markers of disease activity (ANA, anti-dsDNA and complement levels). Half of the patients with lupus nephritis achieved renal remission by 6 months. Overall safety profile seems acceptable with 5/16 patients developing grade III infections; no malignancies or deaths were reported during the 28 months follow up (21). One SLE patient with autoimmune hemolytic anemia who failed rituximab, achieved clinical remission after ofatumumab (22). Four patients with nephritis achieved reduction of proteinuria and anti-dsDNA levels (23). There are no active formal clinical trials in SLE. A 52-week, phase II trial studying safety and efficacy of obinutuzumab, a different anti-CD20 antibody, in lupus neprhritis is currently active with estimated completion date in December 2019 (NCT02550652).

As per EULAR recommendations, anti-CD20 treatment can be tried in refractory SLE patients. Severe infections remain a concern as many of these patients are already receiving other immunosuppressive medications.

### Anti-CD22

CD22 is a surface molecule that modulates B cell activation and migration. Epratuzumab is a humanized anti-CD22 antibody, initially showed positive results reaching its primary endpoint (BICLA response) in the EMBLEM phase II trials (24). However, the beneficial effect was not replicated in the larger and more stringently performed phase III EMBODY trial (25). The reason of failure was thought due to sub-optimal dosing, high placebo response rates, and inadequate optimization of standard of care. Interestingly, some promising response was observed in subgroups of patient with features of Sjogren's syndrome and positive anti-SSA antibodies. Further research is needed to explore this and other potential sub-groups that might respond (26).

### Anti-CD19

CD19 is a surface receptor found exclusively on B cells. XmAb5871 is an antibody that co-engages CD19 and the inhibitory FcγRIIb receptor, resulting in B cell inhibition but not ablation (27). There is an ongoing randomized, double-blinded, placebo-controlled study of XmAb5871 to determine its ability to maintain SLE remission achieved by a brief course of steroid therapy (NCT02725515). This clinical trial has an interesting design: Moderately active SLE patients are taken off traditional therapies and are given high dose parenteral steroids. In theory most patients will become clinically inactive. Then the patients of both XmAb5871 and placebo group are observed for development of flare. The outcome measure would be time to relapse after the initial induction of remission. This unconventional study design is likely to reduce the placebo response by eliminating the effect of background therapy.

### Proteasome inhibitors

The lack of expression of CD20 on plasma cells, especially long-lived plasma cells may be one of the reasons for the poor performance of anti-CD20 therapies in SLE (28). This can be addressed by targeting specifically the plasma cell ability to produce immune globulins by inhibiting the proteasome. This organelle handles misfolded proteins, produced at high levels during immune-globulin assembly, and has proven critical for plasma cell function.

Bortezomib is a proteasome inhibitor, that is efficacious in plasma cell cancers. In lupus prone MPL/lpr mice, administration of bortezomib was found effective in preventing and more importantly treating established disease (29). Similar effects were observed with two other proteasome inhibitors, carfilzomib (30), and delanzomib (31) when used in preclinical models of lupus nephritis (NZBW F1 and MRL/lpr mice). Following these successful studies, bortezomib was infused in 12 patients with refractory SLE. The patients had significant improvement in several clinical parameters such as dermatitis, proteinuria, arthritis, and serositis. Neuropathy developed in 2/12 patients as a major side effect. Currently, bortezomib is assessed in a phase II clinical trial in SLE patients (NCT02102594) with estimated completion date in December 2018.

Although proteasome inhibition is attractive, the main concern for adding these seemingly potent medications in the SLE therapeutic armamentarium remains the severe toxicity associated with their chronic use.

## Intracellular signaling

### Bruton's tyrosine kinase (Btk)

Btk is a B-cell receptor (BCR) associated kinase that activates the NFkB pathway (32). Importantly though it also associates with the Fc receptor in monocytes (33) and can bridge BCR and TLR9 signaling (34). Mutations in the *Btk* gene result in agammaglobulinemia (35). Ibrutinib is already in use for B cell malignancies and has good safety profile (36). In mouse models of lupus nephritis treatment with Btk inhibitors PF-06250112 (37) and ibrutinib (38) resulted in less severe nephritis. This served as the base for the Btk inhibitor MSC2364447C (M2951) to be evaluated in a phase Ib trial in SLE patients with mild to moderate disease (NCT02537028). Fenebrutinib (GDC-0853), an orally available inhibitor of Btk, is evaluated in a phase II clinical study in patients with moderate to severe active SLE (NCT03407482). Participants will receive GDC-0853 twice daily for 48 weeks and will be followed for additional 8 weeks to evaluate the long-term safety and efficacy.

### Cereblon modulator (CC-220)

CC-220 binds to cereblon (CRBN), a substrate receptor of CUL4^CRBN^ E3 ubiquitin ligase complex. As an immunomodulatory compound, CC-220 can lead a substrate specific ubiquitination of transcription factors Ikaros (IKZF1) and Aiolos (IKZF3) both essential for antibody production (38, 40). A Pilot phase II randomized, placebo-controlled, double-blind study is underway to evaluate efficacy, safety, tolerability, pharmacokinetics of CC-220 in patients with SLE (NCT02185040). CC-220 showed some efficacy but there were important safety issues in a 12-week, phase II, dose-escalation study of 42 patients and 14% of patients stopped treatment because of adverse effects. Higher doses of CC-220 were associated with neutropenia, pneumonia, and dermatitis (41).

### Calcineurin inhibitors

Activated lupus T cells show an exaggerated calcium response, which leads to early and sustained activation of the phosphatase calcineurin and its substrate, the transcription factor nuclear factor of activated T cells (NFAT). NFAT upregulates a number of genes, including CD154 (also called CD40L) (42), a critical molecule for T:B cell interaction. Calcineurin inhibitors cyclosporine and tacrolimus, have been successfully used in preventing transplant rejection through blocking this important pathway. Moreover, calcineurin inhibitors may have an antiproteinuric effect, rendering them an important treatment alternative or adjuvant therapy for lupus nephritis. Compared to mycophenolate mofetil, tacrolimus was found to be non-inferior in induction of remission (62 vs. 59%). However, there was a trend for more flares in the tacrolimus group (43).

Voclosporin, a novel calcineurin inhibitor, was investigated in a phase II trial for lupus nephritis as a combination therapy with mycophenolate mofetil (AURA trial). Patients received mycophenolate alone or Voclosporin at 39.5 or 27.5 mg combined with mycophenolate. Remission rates at 6 months favored the combination therapy over mycophenolate alone (OR = 2.03). Unfortunately, the voclosporin-mycophenolate combination resulted in severe side effects including 12 deaths vs. 1 death in the mycophenolate group alone (44) despite the use of rather low corticosteroid doses. Double-blind, placebo-controlled AURORA (NCT03021499) phase 3 clinical trial has started with a plan to include 320 patients with nephritis. It will determine if a combination of voclosporin and a standard of care therapy with mycophenolate mofetil increases kidney function, compared with to standard of therapy alone.

Overall, calcineurin inhibitors alone or combined with mycophenolate represent acceptable alternatives for lupus nephritis treatment; the combination though may carry a significant risk for serious infections.

### Mammalian target of rapamycin (mTOR) signaling inhibitor

mTOR is a highly conserved serine/threonine kinase and is well known to be essential for the regulation of cell metabolism, growth, and proliferation (45, 46). Activated mTOR in lupus T cells is associated with several abnormalities including expansion of both T_H_17 and CD3^+^CD4^−^CD8^−^ double negative T, as well as, contraction of Tregs (47, 48).

Administration of the mTOR inhibitor rapamycin, results in immediate inhibition of mTORC1 signaling and delayed inhibition of mTORC2 signaling. It has a marked effect on the immune system, partly by interrupting metabolic demands associated with lymphocyte proliferation and effector function. In the context of SLE, rapamycin ameliorated nephritis and improved IL-2 production in MRL/lpr mice (49). In an open-label clinical trial, rapamycin improved the clinical and laboratory parameters in patients with recalcitrant SLE (50). A larger clinical trial in SLE (NCT00779194) is under way.

The N-acetylcysteine (NAC), a potent anti-oxidant and glutathione precursor, also inhibits mTOR. NAC administration in SLE patients resulted in improvement of disease activity (51). NAC also led to the expansion of CD4^+^CD25^+^FoxP3^+^ Tregs and depletion of phospho-S6RPhi DN T cells (52). NAC is well tolerated with its major side-effect being nausea at high doses (over 4.8 g per day).

### JAK/STAT inhibitors

The Janus kinase (JAK)/signal transducer and activator of transcription (STAT) system fine-tunes immune cell activation (53), defining their differentiation. In SLE, there is mounting evidence of the critical involvement of this system in disease pathogenesis. In a recent study, it was shown that increased STAT5 signaling in lupus T cells is related to changes in circulating CD4 T cell subsets and correlated with more aggressive disease (54).

Following the success in treating RA with tofacitinib, the first oral JAK inhibitor (55), several JAK inhibitors are currently under investigation. As type I interferons, known to be upregulated in SLE, transduce their signal through JAK, the JAK1 inhibitor GSK2586184 was used in a small trial to block the expression of interferon-related genes in SLE. The trial failed to show a difference (56) without being powered to address the broader effect JAK inhibition may have on disease activity.

Tofacitinib was studied in phase Ib trial in patients with mild to moderate lupus, stratified based on the presence or absence of STAT4 risk alleles. Although data are not available to date, this study is one of the first to address the link between genetic susceptibility and response to treatment in SLE. Baricitinib, a more selective JAK 1/2 inhibitor was evaluated in a phase II trial in SLE patients that was completed with positive yet modest results (57). A third JAK1 inhibitor, Filgotinib, is currently being evaluated in patients with moderate to severe active cutaneous lupus (NCT03134222) and with membranous lupus nephropathy (NCT03285711).

BMS-986165 is broad inhibitor against a panel of 265 kinases and pseudokinases. BMS-986165 protected NZB/W lupus-prone mice from nephritis possibly through its effect on interferon signaling (58). BMS-986165 also suppresses IL-23/IL-17 and IL-12. An ongoing phase 2 randomized, double-blind, placebo-controlled trial is exploring the efficacy and safety in patients with SLE (59). Finally, our group identified STAT3 that associates with JAK and mediates IL-6 and IL-23 signaling, as a potential therapeutic target in SLE (60, 61). STAT3 influences SLE T cell cytokine production, cell migration and B cell activity in lupus prone mice (62). In a preclinical study, Stattic, a small molecular STAT3 blocker, alleviated nephritis in lupus prone MRL/lpr mice (58, 63).

The Jak-STAT pathway therefore represents a very promising therapeutic target in both non-renal and renal lupus. Moreover, the use of small molecular oral agents to inhibit this pathway as opposed to biologic inhibitors of cytokines, makes this approach even more appealing.

### Rho kinase (ROCK) inhibitors

ROCKs are a family of serine-threonine kinases (59) that function as downstream effectors for the GTPase Rho. The main isoforms ROCK1 and ROCK2 regulate multiple biological functions, including proliferation, differentiation, and migration by cytoskeletal reorganization. The potential of this pathway as a treatment target in SLE, was first shown by the ROCK inhibitor Y27632 that blocked the ability of SLE T cells to migrate *in vitro* (64, 65). Subsequently, ROCK2 was found to be selectively activated in murine lupus T cells. In these lupus models, ROCK2 regulates IRF4 and increases IL-17 and IL-21 production (66).

A wide array of available ROCK inhibitors has been investigated in SLE including Fasudil and Y27632. Fasudil is non-isoform selective ROCK inhibitor that attenuates disease activity in MRL/lpr mice and NZBWF1 mice (64). Fasudil can also cause vasodilation and hence was evaluated for the treatment of systemic sclerosis patients with Raynaud's phenomenon. A single oral dose of 40 or 80 mg fasudil though, did not improve skin temperature recovery or increase digital blood flow (67).

Although, there are no current clinical trials investigating ROCK inhibitors in patient with SLE, they are evaluated in patients with angina pectoris, pulmonary hypertension (68), idiopathic pulmonary fibrosis (NCT02688647) and psoriasis vulgaris (NCT02317627).

## Co-stimulation

T cell activation is a tightly controlled process that consists of several steps to allow proper T cell differentiation. Each step in this process has the potential to serve as therapeutic target for autoimmune diseases. Following antigenic binding to the T-cell receptor, the strength of the immune response depends on the expression and interaction of costimulatory surface molecules on antigen presenting cells with those on T cells (68, 69). Here we review the role of disrupting the two most important co-stimulatory pairs, CD28/B7 and CD40/CD154 as a therapeutic strategy in SLE.

### CD28/B7

Inhibition of co-stimulatory pathway has already been utilized in in the treatment of rheumatoid arthritis with abatacept. This is a fusion protein made of CTLA4 (cytotoxic T-lymphocyte-associated protein 4) and an immunoglobulin chain (CTLA4-Ig). It binds CD80/86 with a higher affinity than CD28. This interaction leads to both inhibition of T cell proliferation and B cell antibody production (70, 71). Efficacy and safety of abatacept added to standard of care with mycophenolate mofetil and steroids was evaluated in a phase III double-blind placebo-controlled trial that randomized over 400 lupus patients with class III or IV nephritis. The study end point was complete renal remission and corticosteroid dose assessed at 52 weeks. The trial did not reach its primary endpoint with 35% of patients treated with abatacept achieving remission vs. 33% in the placebo group (*p* = 0.73). Abatacept treatment was associated with improvement of immunologic markers (anti-ds DNA, C3, and C4 levels) as compared to placebo. In patients with nephrotic-range proteinuria, treatment with abatacept led to more rapid and greater reduction of proteinuria compared with placebo. Infection rates were similar as previously reported for RA patients. Thus, far, there have been several other trials of abatacept in active lupus nephritis but none achieved its primary endpoint (72, 73).

Targeting the CD28 instead of B7 has been challenging by the lack of inhibitory antibodies that would not crosslink CD28 and trigger a cytokine storm (74). One solution was to develop pegylated monovalent anti-CD28 antibodies such as lulizumab (75). Lulizumab was evaluated in phase II trial in non-renal lupus following acceptable safety profile in phase I trial. The trial was terminated early as it failed to meet protocol objectives (NCT02265744).

### CD154/CD40

Another pair of co-stimulatory receptor-ligand system whose engagement has profound effects on B, dendritic and endothelial cells is the CD40-CD40 ligand (CD154). Following T cell activation, CD154 is expressed on the surface of the cell, allowing binding to B cells through CD40; that in turn leads to IgG class switching (76). Two drugs are currently explored as therapeutic agents in phase II trials for patients with moderately to severely active SLE. The first one, Dapirolizumab, a polyethylene glycol conjugated anti-CD40L Fab' fragment, was well tolerated in phase I study (77). At 12 weeks of treatment 46% of high disease activity patients showed reduction in disease activity measured by BILAG and 41% had improved SRI-4. The second one, BI 655064 is a humanized monoclonal anti-CD40 antibody. Its efficacy will be assessed in a double-blind, randomized, placebo-controlled trial for patients with active class III and IV lupus nephritis (NCT03385564). The study is actively enrolling and the primary endpoint is defined as complete renal response at 1 year.

## Cytokines

SLE is characterized by skewed cytokine production that can directly cause local tissue damage and contribute to systemic symptomatology. Besides the interferons, other inflammatory and immunomodulatory cytokines have been investigated as therapeutic targets for SLE.

### Interleukin-2 (IL-2)

There has been resurgent interest in interleukin-2 since the discovery of its homeostatic potential on CD4 T cells and its ability to redirect immune responses toward tolerance. IL-2 promotes the expansion and survival of regulatory T cells, and can be used in low doses to promote tolerance averting graft vs. host disease in bone marrow recipients (78–80). In SLE, low-dose IL-2 therapy is of particular interest, as these patients have low levels of IL-2, defective regulatory T cell function, and overactive T effector cells (81, 82). This imbalance can potentially be reversed with the addition of IL-2 (83). In preclinical studies, low dose IL-2 abrogated the development of nephritis in lupus-prone mice and mediated selective expansion of regulatory T cells in SLE patients (84, 85). This approach was tested in an open label phase I/II trial of subcutaneous low-dose IL-2 injection on alternate days for 3 cycles in 38 SLE patients. More than 80% of patients showed significant SRI-4 response by week 12, in addition to increased numbers of regulatory T cells, decreased Th17, follicular helper and double negative T cells (86, 87). This paved the way for larger placebo-controlled trials of low dose IL-2, using different IL-2 preparations and dosing schedules: AMG 592 (NCT03451422); LUPIL-2 trial with ILT-101 (NCT02955615); and Charact-IL-2 with Aldesleukin (NCT03312335).

### Interleukin (IL)-12/23

Elevated IL-23 levels have been found in patients with lupus nephritis (83, 88, 89). Activation of IL-23/IL-17A axis induces expansion of highly pathogenic T_H_17 cells, ultimately contributing to pathogenesis of lupus nephritis by enhancing immunoglobulin and complement deposition (90, 91). Sole targeting of IL-17 in murine lupus nephritis models either with genetic deletion or utilizing a blocking antibody against IL-17A had no impact on the disease (92, 93). However, upstream targeting of this axis with ustekinumab, a monoclonal anti-IL-12/23 antibody that is already approved and well tolerated in patients with a variety of autoimmune diseases, showed promising data. Ustekinumab was evaluated in placebo-controlled phase II trial that recruited 102 SLE patients with active disease despite ongoing standard of care therapy (steroid, antimalarial and/or immunosuppressive therapies) (94). The protocol allowed intravenous loading, followed by subcutaneous administration every 8 weeks. At week 24, ustekinumab arm showed a significant improvement of the SLEDAI-2K score compared to placebo (SRI-4: 60 vs. 31%, respectively, *p* = 0.0046), which was the predetermined primary endpoint. A number of other metrics also improved, including anti-dsDNA, C3 levels, musculoskeletal and mucocutaneous manifestations. Moreover, a significant lower risk of a new BILAG flare was found in the ustekinumab group (*p* = 0.0078) but there was no difference in BILAG or BICLA scores at week 24 among the groups. Safety and adverse events of ustekinumab were similar to safety profile reported for other indications. Overall, this is very promising therapeutic option with an ongoing phase III trial that will address ultimately its usefulness in SLE.

### IL-10

IL-10 has been shown to be increased in the serum of SLE patients and levels do correlate with disease activity (95). Its exact role in the propagation of the disease is unclear as IL-10 has both pro and anti-inflammatory effects. The anti-IL-10 antibody, BT063, is currently undergoing a phase II trial (NCT02554019); the trial aims at recruiting 36 patients with SLE who are to receive 50 mg of BT063. Safety and efficacy will be compared to standard of care. The drug will be administered over 8 cycles of intravenous infusion in 12 weeks period. Although no data are available, the clinical development program for this molecule is active.

### IL-6

IL-6 is a proinflammatory cytokine found to be elevated in patients with active SLE (96). Rationale for its therapeutic blockade comes from data that showed diverse biologic function, spanning from promoting terminal differentiation of B and T_H_17 cells to locally driving tissue damage (97–99). Additionally, in animal models of SLE, disrupting IL-6 signaling either by utilizing an anti-IL-6 monoclonal antibody or anti-IL-6 receptor antibody led to improved survival, decreased levels of ds-DNA and proteinuria (100, 101). The opposite was found when mice were injected with recombinant human IL-6 (101, 102). Unfortunately, the clinical trials though have not been as encouraging.

PF-04236921, a monoclonal IL-6 antibody failed to meet its primary efficacy endpoint (SRI-4) a phase II trial that enrolled 183 patients assigned to receive subcutaneous 10 mg, 50 mg or 200 mg drug or placebo (103). In the 200 mg dose group, there were four deaths secondary to infections and thrombosis. A subgroup analysis showed that benefit can be seen in patients with high disease activity at baseline who received the 10 mg dose. They had significantly improved SRI-4 and BICLA response rates compared to placebo (49 vs. 25.1%, *p* < 0.05) and decreased incidence of severe lupus flares. Two other monoclonal antibodies failed to demonstrate efficacy in phase II trials, sirukumab (104), and vobarilizumab. Finally, the monoclonal antibody MRA003US is currently in a phase I trial (NCT00046774) and no results have been released to date.

### The interferons (IFN)

The hypothesis that IFNs have an important role in SLE pathogenesis is supported by plethora of findings both in humans and animals. Patients with active lupus have elevated levels of type I IFN. Moreover, patients with active and quiescent disease have evidence of continuous exposure to type I interferons based on multiple gene expression studies that show upregulated interferon responsive genes, collectively known as “IFN signature” (105–108). Given modulatory potential of IFNs to initiate or amplify immune responses leading to organ damage in lupus, this cytokine system became an excellent therapeutic target.

Sifalimumab, an anti-IFNα monoclonal antibody was evaluated in a phase II clinical trial in patients with moderate to severe SLE (109). Compared to placebo, patients receiving monthly IV infusions of sifalimumab (1,200, 600, and 200 mg groups) had statistically superior SRI-4 response index compared to placebo that received standard of care treatment at 52 weeks (sifalimumab: 59.8, 56.5, and 58.3 vs. 45.4% placebo, respectfully). At baseline, approximately 80% of patients had the IFN signature of gene expression at baseline, and tended to have better responses. The most common infectious complication was herpes zoster in patients receiving high dose (9.3 vs. 0.9% in the placebo group) that responded to treatment. There was one recurrence among patients who continued receiving sifalimumab. Overall, this was a positive study but given the modest effect size, there was no further development of this drug.

Rontalizumab is another humanized IgG1 anti-IFNα antibody that can neutralize all 12 subtypes of interferon-alpha. It was evaluated in the placebo control phase II trial, ROSE (110). At baseline, 76% of patients had high IFN regulated gene expression. At 24 weeks of treatment, the drug failed to meet the primary endpoint. Unexpectedly, treatment with rontalizumab showed consistent benefits with higher SRI-4 responses compared to controls in the subgroup of patients who had low interferon signature detected (72.7 vs. 41.7% placebo) and this group achieved meaningful rates of prednisone dose reduction to ≤10 mg daily. Rontalizumab was well tolerated and serious adverse events were 14.6 vs. 8.3% in the placebo group, all classified as unrelated to the study drug. Therefore, higher doses of rontalizumab would be well tolerated and possibly more effective but no additional trials are planned to answer this question. There are currently two phase I trials with other monoclonal antibodies against IFN-α: IAGS-009 completed phase I (NCT00960362) and JNJ-55920839 is in recruiting phase (NCT02609789).

Another strategy to inhibit the IFN-I pathway is to block its receptor, the interferon alpha receptor 1 (IFNAR1) that binds all type I IFNs including IFNα and IFNβ. Anifrolumab, a monoclonal antibody against IFNAR1, was granted fast- track status by the FDA and was successful in phase II open-label trial (108, 109). In this study, 75% of patients had high IFN signature at baseline. The primary endpoint consisting of composite SRI-4 combined with a measure of steroid sparing to <10 mg/day was achieved in both anifrolumab dose groups at higher rates than placebo (28.8% in the 1,000 mg IV monthly, 34.3% in 300 mg IV monthly vs. 17.6% in the placebo group). At 1 year, 56.4% of the patients taking a 300 mg dose met the SRI end-point, as compared to 31.7% receiving 1000 mg dose (*p* = 0.595) and 26.6% on placebo. Regarding the infections rate, patients receiving anifrolumab had dose-dependent increase in herpes zoster cases (placebo: 2.0%; 300 mg: 5.1%; 1,000 mg: 9.5%) and a greater number of influenza infections (placebo: 2.0%; 300 mg: 6.1%; 1,000 mg: 7.6%). However, most cases of influenza were unconfirmed. With this positive data, two phase III studies are currently underway via the TULIP (NCT02547922) program. TULIP-LN1 on the other hand is phase IIb study designed to assess efficacy and safety of two intravenous doses of anifrolumab vs. placebo while taking standard of care treatment with mycophenolate mofetil and corticosteroids in adults with active proliferative lupus nephritis. On August 31, 2018, it was reported that in one of the phase III trials (TULIP I), anifrolumab failed to meet its primary endpoint.

In conclusion, IFNAR blocking has been a promising therapeutic approach for SLE patients who fail to respond to available therapies. The recently released result though from the phase III trial dampen the enthusiasm for the usefulness of this approach.

### Interferon-γ (IFNγ)

The pathogenic role of IFNγ has been better characterized in mice, as opposed to humans (111), where elevated levels and correlation with disease activity is found in both NZB/W and MRL/lpr mice. Administration of IFN-γ accelerates murine lupus while early treatment with anti-IFNγ antibody rescues mice from disease (112). Unfortunately two phase I studies of the anti-IFNγ antibody AMG811 in the treatment of mild to moderate systemic and cutaneous lupus (113) showed safety with favorable immunogenicity profile but did not show significant therapeutic effect despite decreasing IFNγ-related gene expression (114). The negative results from these small studies led to discontinuation of the development program for AMG811.

### IFNα kinoid (IFNα-K)

This is another interesting approach for neutralizing I IFN that received fast track status by the FDA. It is currently in phase IIb trial aiming at recruiting 185 patients (NCT02665364). Patients are assigned to receive IFNα-K immunotherapy or placebo in addition to standard treatment with immunosuppressives, antimalarials, and/or steroids. The drug is composed of inactivated IFN-α coupled to the keyhole limpet haemocyanin protein and when injected, leads to induction of polyclonal anti-IFNα responses with transient immunity against all 13 subtypes of interferon alpha. In preclinical stage, IFNα-K was able to slow disease progression in NZB/W mice (115). From an infectious standpoint, this would be an advantageous approach as cellular tolerance and host defense against viral infections would remain intact. The primary end point in the phase II is the BILAG-based Composite Lupus Assessment (BICLA) response at week 36.

### Other

Lupuzor/P140 peptide or regiremod, is a 21-mer linear peptide derived from nuclear ribonucleoprotein U1-70K that needs to be phosphorylated at the Ser140 position in order to exert its immunomodulatory properties via binding to MHC class II. This allows recognition in the context of T cell receptor, both in lupus patients and mice and alters autoreactive T cell phenotype. In a phase IIb data, patients receiving Lupuzor 200 μg subcutaneously every 4 weeks achieved the SRI response at week 12 at higher rates than standard of care therapy that included stroids, antimalarials, azathioprine or methotrexate (53.1 vs. 36.2%). These positive results in IIb trial were more pronounced among patients with SLEDAI-2K ≥6 at baseline, showing that 61.9% achieved SRI response at week 12 vs. 38.6% in the placebo group. Nevertheless, in the phase III clinical trial, Lupuzor failed to meet the primary endpoint (*p* = 0.2631 vs. placebo). The investigational treatment in this trial though holds promise for patients with anti-dsDNA autoantibodies as 7.6% of these patients in the Lupuzor group went into full remission, compared with none in the placebo treated group. The company launched 6 months open label extension study for all participants of phase III trial, allowing continuation of Lupuzor treatment in combination with standard therapy for additional 48 weeks. Finally, this study confirmed the already known Lupuzor's good safety profile with zero adverse effects reported.

## Conclusions

To date, belimumab (anti-BLyS) is the only FDA approved biologic for treating SLE. Over the last decade and despite the setbacks including the recent failure of the highly promising anti-IFNαR therapy, our understanding of the mechanisms of SLE contributed to expansion of the drug pipeline for SLE (Table 1). Currently, drugs representing a variety of therapeutic strategies are moving to phase III trials. These include: cytokine infusions (low dose IL-2); antibodies against cytokines (ustekinumab); and finally, small molecule inhibitors against kinases (Jak inhibitors) and phosphatases (calcineurin inhibitors). It is highly likely that these targeted therapies in conjunction with biomarker development and more rigorous outcome measures will finally result in a fundamental change of the stagnant therapeutic paradigm in SLE.

**Table 1:** New and emerging therapies in SLE.

**Molecular target**	**Treatment**	**Status**
**B CELLS**		
BAFF/APRIL	Belimumab	Approved for non-renal SLE Ongoing phase IV for efficacy, safety, and tolerability Ongoing phase III in combination with Rituximab
	Tabalumab	Phase III without significant effect (terminated)
	Blisibimod	Phase III did not meet SRI-6 primary end point
	Atacicept	APRIL-SLE study terminated due to increased infection rate ADDRESS II study has acceptable safety profile
CD20	Rituximab	Phase III failed (nephritis and non-nephritis)
	Ocrelizumab	Phase III trial completed
CD22	Epratuzumab	Phase III failed
CD19	XmAb5871	Phase II trial
Proteasome inhibitors	Bortezomib	Phase II trial
**INTRACELLULAR SIGNALING**		
Btk	M2951	Ongoing phase II
	Fenebrutinib	Ongoing phase II trial
mTOR	N-acetylcysteine	Small study showed decrease in SLEDAI, no further development
	Rapamycin	Open-label study showed an effect on BILAG. Larger study planned.
JAK/STAT	GSK2586184	Ineffective on interferon signature in phase II, safety data do not support further study
JAK 2	Baricitinib	Phase II positive data; Phase III trial ongoing
JAK3	Tofacitinib	Ongoing Phase I/II trial
ROCK	Fasudil	Effective in preclinical studies in patient with Raynaud's, phase III completed with uninterpretable data.
**CO-STIMULATION**		
CD40:CD154	Dapirolizumab	Ongoing phase II trial
	BI 655064	Ongoing phase II trial
CD28:B7	Abatacept	Ineffective in phase III in nephritis and general SLE
	Lulizumab	Phase II trial terminated—failed to meet protocol objectives
**CYTOKINES**		
	Sifalimumab	Limited effect in phase II and III. No further development
	Rontalizumab	Phase II without significant results
Interferon-α	Anifrolumab	Phase II positive data; 2 Phase III trials ongoing (one reported negative)
	IAGS-009	Completed phase I, no data released
	JNJ-55920839	In recruiting phase
	IFNα-k	Successful phase I; ongoing phase II trial
Interleukin-2	Aldesleukin	Ongoing open-label phase II trial
	AMG 592	Ongoing phase Ib and IIa trial
	ILT-101	Ongoing phase II trial
Interleukin 12/23	Ustekinumab	Met primary end-point in phase II trial; ongoing phase III trial
Interleukin-6	PF-04236921	Failed phase II trial; safety compromised
	Sirukumab	Failed phase II trial
	MRA003US	Ongoing phase II trial
	Vobarilizumab	Ongoing phase I trial
Interleukin-10	BT063	Ongoing phase II trial
**OTHER**		
	Lupuzor	Phase III trial failed to meet the primary end point

## Author contributions

All authors listed have made a substantial, direct and intellectual contribution to the work, and approved it for publication.

## Conflict of interest statement

VK is a site PI for the EMD Serono sponsored phase II, study to evaluate the safety and efficacy of M2951 in subjects with SLE. The remaining authors declare that the research was conducted in the absence of any commercial or financial relationships that could be construed as a potential conflict of interest.
